# The Proportion of Fermented Milk in Dehydrated Fermented Milk–Parboiled Wheat Composites Significantly Affects Their Composition, Pasting Behaviour, and Flow Properties on Reconstitution

**DOI:** 10.3390/foods7070113

**Published:** 2018-07-14

**Authors:** Ashwini V. Shevade, Yvonne C. O’Callaghan, Nora M. O’Brien, Tom P. O’Connor, Timothy P. Guinee

**Affiliations:** 1 Teagasc Food Research Center, Moorepark, Fermoy, Co. Cork P61 C996, Ireland; ashwini.shevade@teagasc.ie; 2 School of Food and Nutritional Sciences, University College Cork, Cork T12 Y337, Ireland; y.ocallaghan@ucc.ie (Y.C.O.); nob@ucc.ie (N.M.O.); tpoc@ucc.ie (T.P.O.)

**Keywords:** fermented milk, parboiled wheat, dehydrated composite, composition, reconstitution, pasting behavior, flow properties

## Abstract

Dairy and cereal are frequently combined to create composite foods with enhanced nutritional benefits. Dehydrated fermented milk–wheat composites (FMWC) were prepared by blending fermented milk (FM) and parboiled wheat (W), incubating at 35 °C for 24 h, drying at 46 °C for 48 h, and milling to 1 mm. Increasing the weight ratio of FM to W from 1.5 to 4.0 resulted in reductions in total solids (from 96 to 92%) and starch (from 52 to 39%), and increases in protein (15.2–18.9%), fat (3.7–5.9%), lactose (6.4–11.4%), and lactic acid (2.7–4.2%). FMWC need to be reconstituted prior to consumption. The water-holding capacity, pasting viscosity, and setback viscosity of the reconstituted FMWC (16.7% total solids) decreased with the ratio of FM to W. The reconstituted FMWC exhibited pseudoplastic flow behaviour on shearing from 18 to 120 s^−1^. Increasing the FM:W ratio coincided with a lower yield stress, consistency index, and viscosity at 120 s^−1^. The results demonstrate the critical impact of the FM:W ratio on the composition, pasting behavior, and consistency of the reconstituted FMWC. The difference in consistency associated with varying the FM:W ratio is likely to impact on satiety and nutrient value of the FMWCs.

## 1. Introduction

Dairy products and cereals constitute two of the major food groups [1]. They are frequently combined in many foods, including bakery products, breakfast cereals, soups, nutritional snack biscuits/bars, cereal yoghurts, fortified blended foods (FBFs), kishk, and tarhana [2,3,4]. FBFs are supplied by the World Food Programme [5], and are primarily targeted at children 0.5–2 years in food insecure regions in over 70 countries. A special category of FBF, referred to as Super Cereal Plus, is a dehydrated composite of heat-treated cereal (wheat, maize, or rice), dehulled soybean, sugar, skim milk powder (8%, *w/w*), refined soyabean oil, vitamins, and minerals; consumption involves reconstitution of the powder to 16.7% solids, heating to 95 °C, and simmering until the desired soup- or porridge-like consistency is attained [6].

Kishk and tarhana are traditional dehydrated fermented milk–cereal based products that are widely available in the Middle East, where they are typically consumed in reconstituted form as a nutritious porridge or soup [2,7,8,9], but also used as an ingredient in an array of dishes such as Lebanese kishk pie, Lebanese Manakeesh, biscuits, and sweet dishes. Kishk is typically formulated from fermented milk (yoghurt) and parboiled wheat (bulgur), whereas tarhana generally contains wheat flour instead of parboiled wheat, and additionally contains yeast, cooked vegetables, and spices [9]. Semi-liquid foods, such as porridge and soup, are an important nutrient vector for infants and young children [10]. An increase in the viscosity of such foods lowers hunger, delays gastric emptying, and increases satiety [11,12,13]. An increase in the protein content of such foods prolongs the feeling of fullness. The addition of milk to cereal enhances the content of essential amino acids and fat-soluble vitamins in the end product [9]. Moreover, the use of fermented milk rather than milk or dehydrated skim milk powder (as in Super Cereal Plus) improves the potential nutritional value of dairy–cereal blends by increasing the content of bioactive compounds [14], reducing the content of anti-nutrients, such as phytic acid, and thereby increasing mineral bioavailability [10,15,16]. Apart from the potential benefits associated with fermentation, the relatively high proportion of milk solids in kishk (37% of total) contributes to a high concentration of calcium, an essential nutrient required for bone and dental health [17]. Hence, the formulation of FBF, based on the use of fermented milk cereal, as in kishk, is likely to be nutritionally advantageous when compared to the inclusion of a lower content of non-fermented milk solids, as in Super Cereal Plus.

A survey of commercial Lebanese kishk powders indicated wide variation in composition, with starch ranging from 40 to 60%, protein from 14 to 21%, fat from 2 to 11%, and salt from 1.0 to 4.5% [18]. Various studies have evaluated the effects of formulation (e.g., different cereals, type of starter culture used in milk fermentation, substitution of yogurt with kefir) on the compositional, microbiological, nutritional, and sensory characteristics of laboratory-made kishk [8,19,20,21,22,23,24]. To our knowledge, no information is available on the viscosity/consistency properties of reconstituted kishk, or how they are affected by the ratio of fermented milk to cereal (FMC ratio); the latter would alter the starch content, and therefore, most likely the consistency, which is an important sensory characteristic [4,19]. In contrast, various studies have examined the rheological properties of reconstituted tarhana. Ibanoğlu and Ibanoğlu [25] evaluated the viscosity of tarhana soup, prepared by reconstitution of the powder to 10–12% solids and heating at 90–100 °C for 5–10 min. Viscosity increased as the powder particle size was reduced from 1000 μm to 500 μm, and decreased with increasing shear rate (500–2000 s^−1^) and measuring temperature (30–70 °C). Bilgiçli [26] reported that the viscosity of tarhana soup decreased linearly as the ratio of buckwheat flour to wheat flour was increased stepwise from 00:100 to 100:00. Yilmaz et al. [27] found that the addition of whey concentrate influenced the viscosity and shear thinning behaviour of tarhana soup to a degree dependent on the quantity added and temperature of measurement.

The objective of the current study was to investigate the effects of altering the ratio of fermented milk to parboiled wheat on the composition, pasting characteristics, and rheological characteristics of reconstituted fermented milk-wheat composites (FMWC). The study formed part of a larger project entitled “Development of Fortified Blended Foods using fermented buttermilk/cereal” [28].

## 2. Materials and Methods 

### 2.1. Ingredients Used

Wheat starch (Amidon de Ble), containing 82.86% starch, was kindly supplied by Roquette Frères (Lestrem, France). Wheat (*Triticum aestivum* sparrow) was donated by Goldcrop Ltd (Springhill, Carrigtwohill, Cork, Ireland). Samples of parboiled and non-parboiled wheat were prepared as described below. Buttermilk powder (BMP; protein 33%, fat 7%, lactose 46%, lactic acid 0.23%) was obtained from Glanbia Ingredients Plc., Ballyragget, Co. Kilkenny, Ireland. Low-heat skim milk powder (SMP; protein 38.43%, fat 0.89%, lactose 46.2%, lactic acid 0.04%) was manufactured using a pilot-scale NIRO Tall-Form Dryer in Moorepark Technology Limited (Teagasc, Moorepark, Co. Cork, Ireland), as described by Lin et al. [29]. Cream (fat 37%) was purchased from a local retail store.

### 2.2. Preparation of Fermented Milk

BMP and SMP were reconstituted to 7% and 8.9% total solids (TS), respectively, in distilled water and blended at 50 °C for 15 min using a Silverson mixer at 750 rpm (Model AX3, Silverson Machines Ltd., Chesham, UK) and cream was added at a level 4.5%. The recombined milk (30 L) was cooled to 15 °C, held overnight at 5 °C to ensure protein hydration [30], heat-treated at 95 °C for 2.5 min, homogenized at first- and second-stage pressures of 15 and 5 MPa, respectively, and cooled to 43 °C (UHT/HTSTLab-25 EHVH, MicroThermics^®^, Raleigh, NC, USA). The contents of TS, protein, fat, and lactose in the recombined milk were 16.8%, 5.6%, 2.1%, and 7.4% (*w*/*w*), respectively.

The milk was sub-divided into 5 L quantities, each of which was inoculated at the recommended level with direct-vat starter cultures CH1 YoFlex^®^ 207 (*Streptococcus thermophilus*) and YC380 (*Lactobacillus delbrueckii* subsp. *bulgaricus*), at a weight ratio of three (Chr. Hansen Ireland Ltd, Rohan Industrial Estate, Little Island, Co. Cork, Ireland). The inoculated milk was incubated at 42 °C for 4–5 h (Heratherm™ Advance Protocol Microbiological Incubators, Thermo Scientific™, Waltham, MA, USA) until the pH decreased to 4.6, at which time the fermented milk had gelled. The FM milk was cooled to 15 °C in ice water while stirring at 120 rpm (Model RW 16, IKA Werke GmbH, Staufen im Breisgau, Germany) and stored at 4 °C overnight.

### 2.3. Preparation of Parboiled Wheat

Wheat (10 kg) was dehusked using a compressed air huller (Streckel Anlagenbau & Verfahrenstechnik GmbH, Hamburg, Germany) and parboiled by blending with water (1.5 kg water/kg dehusked kernel) and heating to 90 °C (Ceran 500 plate, Harry Gestigkeit GmbH, Germany) for 60–70 min, until all water was visually absorbed. The parboiled cereal was spread out in layers (30 cm × 30 cm × 0.5–1.0 cm) on ParaFlexx Premium Nonstick solid drying sheets, dried at 52 °C (Excalibur^®^ Dehydrator, Sacramento, CA, USA) for 24 h to 6–7% moisture, and milled to a mesh size of 1 mm (Ultracentrifugal Mill ZM 200, Retsch Technology GmbH, Haan, Germany). The contents of TS, protein, fat, and starch of the parboiled wheat were 93.4%, 11.2%, 2.2%, and 59.2% (*w*/*w*), respectively.

### 2.4. Preparation of Dehydrated Fermented Milk–Wheat Composite

Two trials were undertaken on separate occasions (days), using different batches of FM and parboiled wheat on each occasion. In each trial, the FM and parboiled wheat were blended at 5 different weight ratios of 1.5, 1.9, 2.3, 3.0, or 4.0, and each blend weighing a total of 2 kg was mixed for 5 min (Kenwood blender, Model KMM710 fitted K-beater; Kenwood Ltd., Fareham, Hampshire, UK). The blends were denoted FMWC 1.5, FMWC 1.9, FMWC 2.3, FMWC 3.0, and FMWC 4.0, respectively. In each trial, a second set of salted blends were prepared, where sufficient salt was added to the FM prior mixing with the parboiled wheat to give a concentration of 1% (*w*/*w*) in the final blend. The salted blends were denoted FMWC 1.5s, FMWC 1.9s, FMWC 2.3s, FMWC 3.0s, and FMWC 4.0s. The formulations of the unsalted and salted FMWCs blends are given in Table 1.

The resultant blends were incubated at 35 °C for 24 h (Heratherm Incubator, ThermoFisher Scientific, Waltham, MA, USA), during which time they acquired a dough-like consistency. The dough was manually rolled into layers (0.5–1.0 cm) on Paraflexx premium non-stick drying sheets (30 cm × 30 cm) using a pastry roller, and dried for 48 h at 46 °C (Excalibur^®^ Dehydrator, Sacramento, CA, USA) until the moisture reached 6–7%. The resultant dried cake was manually broken into pieces, size-reduced (Hallde RG-350 machine, AB Hallde Maskiner, Kista, Sweden) and milled (Ultracentrifugal Mill ZM 200 fitted with a trapezoidal 1 mm ring sieve; Retsch Technology GmbH, Haan, Germany) to a powder. The resultant dehydrated FMWC (powder) was vacuum-packed in polythene liners and stored at 15 °C.

### 2.5. Analysis of Dehydrated Fermented Milk–Wheat Composites 

#### 2.5.1. Composition

The FMWC were analysed in triplicate for protein by Leco nitrogen analyser (Model FP268, Leco Corporation, Saint Joseph, MI, USA), fat by CEM Smart Trac (CEM Corporation, Matthews, NC, USA), NaCl by potentiometric determination of chloride [31], and moisture by drying to constant weight at 102 °C [32]. The pH was measured on a 5% aqueous dispersion of the FMWC, prepared by stirring for 15 min at 21 °C (InoLab 7310 pH meter, WTW GmbH, Weilheim, Germany). The concentration of starch was measured by the Megazyme enzymatic K-TSHK 09/15 kit, lactose, and galactose by the Megazyme enzymatic K-LACGAR kit, and lactic acid by the Megazyme enzymatic K-DLATE kit (Megazyme International Ireland, Bray Business Park, Bray, Co. Wicklow, Ireland).

#### 2.5.2. Particle Size

The particle size distribution of the FMWC was measured by laser diffraction, using a Malvern Mastersizer 3000 (Malvern Instruments Ltd., Malvern, United Kingdom) with an automated dry powder Aero S dry dispersion unit, as described by Silva and O’Mahony [33]. The air pressure and powder feed rate were 1.5 bar and 30%, respectively. Particle refractive and adsorption indices were set at 1.45 and 0.1, respectively, and measurements made at an obscuration setting of 6%. Size measurements are recorded as D_10_, D_50_, and D_90_, whereby 10%, 50%, and 90% of the FMWC particles have a diameter less than the value recorded.

#### 2.5.3. Water Sorption

The water desorption and sorption as a function of relatively humidity (RH) in the range 85–5% was measured gravimetrically using the SPS11 automatic multi-sample moisture sorption analyser (Project-e Messtechnik, Enderlegasse, Ulm, Germany), as described by Hogan and O’Callaghan [34]. The FMWC (500 mg) was weighed into aluminium dishes (Sartorius BP211D analytical balance, Satorius AG, Göttingen, Germany), placed in the sorption analyser at 20 °C, and equilibrated at 85% RH. The RH was reduced stepwise from 85% down to 5% and then back to 85%, at intervals of 10% RH. A time of 12 h was allowed for each step change in RH, in order to ensure equilibrium (i.e., weight change less than 0.01% within 40 min). The results are expressed as moisture content per 100 g dry matter of the FMWC, as a function of RH.

### 2.6. Analysis of Reconstituted Fermented Milk-Wheat Composites

#### 2.6.1. Water Holding Capacity 

The FMWC was dispersed to a final TS content of 16.7% (*w*/*w*) in distilled water at 25 °C, while continually stirring at 500 rpm (IKA^®^ RT10 Magnetic Stirrer, IKA-Werke GmbH & Co. KG, Staufen, Baden-Württemberg, Germany) for 3 min. The contents of the beaker were transferred to a doubled-jacketed, capped glass vessel (Therm 500 mL, Product No. 61418250, Metrohm Ireland Ltd., Carlow, Ireland), fitted with an overhead stirrer (Model RW 16, IKA Werke GmbH, Staufen im Breisgau, Germany) and connected to a thermostatically-controlled water bath (Model Julabo EH-5, Julabo GmbH, Seelbach, Germany). The dispersion was stirred at 120 rpm while heating to 95 °C over 10 min, held at 95 °C for 25 min, and cooled to 21 °C over 10 min. The water holding capacity (WHC) was measured in duplicate at 0 (before heating), 10 (after heating to 95 °C) and 35 min (after holding at 95 °C for a further 25 min). The entire contents of the glass vessel were transferred to a 250-mL centrifuge bottle, weighed, and centrifuged at 3000× *g* for 1 h at 20 °C (Sorvall Lynx 6000 Superspeed centrifuge, ThermoElectron LED GmbH, Langenselbold, Germany). The supernatant was decanted, and the weight of the pellet was recorded. The WHC was defined as the weight of the pellet expressed as a percentage of the sample weight before centrifugation, and expressed as g pellet/100 g reconstituted FMWC.

#### 2.6.2. Gelatinization Temperature

Gelatinization temperature was determined using differential scanning calorimetry (DSC 2000, TA instruments, New Castle, DE, USA). Samples (1–3 g) of the FMWC, wheat starch, parboiled wheat, and non-parboiled wheat were reconstituted in distilled water at 20 °C to a fixed water-to-starch ratio of 11.4, and stirred for 15 min at 500 rpm (IKA^®^ RT 10 Magnetic Stirrer, IKA-Werke GmbH, Staufen im Breisgau, Germany). A sub-sample (20–30 mg) was weighed into a Tzero hermetic pan (901683.901, TA Instruments, Flawil, Switzerland), sealed (Tzero 901684 lids), equilibrated at 20 °C, and heated to 95 °C at 5 °C/min. An empty pan was used as a reference. For each sample endotherm, the temperature at gelatinization onset (To), peak (Tp), and end (Te) were obtained using the system software.

#### 2.6.3. Pasting Behaviour

The FMWC was reconstituted to 16.7% TS and pasted on a controlled stress rheometer (Anton Paar Physica MCR 501 Rheometer, Anton Paar GmbH, Graz, Austria), fitted with a starch pasting cell, comprising a measuring cup (CC26/ST; internal diameter, 26 mm) and stirrer (ST24; diameter, 24 mm; length, 122 mm). FMWC (2.5 g) and distilled water (12.5 g) were added to the cup, tempered at 25 °C for 1 min, heated to 95 °C over 10 min, held at 95 °C for 25 min, and cooled to 30 °C over 10 min while constantly shearing at 160 s^−1^. Samples of wheat starch, parboiled wheat, and non-parboiled wheat were also assayed as reference samples. Wheat starch was reconstituted to give starch levels ranging from 6 to 9.0%, and parboiled and non-parboiled wheat to a starch level of 6.3% (equivalent to that in the reconstituted FMWC 4.0).

The analyses were performed in duplicate, and the following parameters were recorded: viscosity after heating to 95 °C over 10 min (V_95_), holding at 95 °C for 25 min (V_h_), and cooling to 30 °C over 10 min (V_c_); and setback viscosity (SBV), which corresponds to the viscosity increase during cooling.

#### 2.6.4. Rheological Properties 

The FMWC was reconstituted to 16.7% TS, heated to 95 °C over 10 min, held at 95 °C for 25 min, and then cooled to 60 °C over 5 min; the temperature of 60 °C was chosen to simulate the temperature at which the cooked reconstituted FMWC power is typically consumed. The flow behaviour of the cooled reconstituted FMWC, on shearing from 18 to 120 s^−1^, was measured on a controlled stress rheometer (Carri-Med, type CSL2500, TA instruments, New Castle, DE, USA), as described by Lin et al. [30]. The sample was subjected to a shear rate sweep at 60 °C, whereby $\dot{\gamma }$ was increased from 18 to 120 s^−1^ over a period of 20 min. Shear stress (σ; Pa) and viscosity (η; Pa∙s) were measured as a function of shear rate, $\dot{\gamma }$. The resultant $\dot{\gamma }$ vs. σ data were fitted to the Herschel–Bulkley model using TA data analysis software (TA Rheology Advance Data Analysis, Version V5.7.0, New Castle, DE, USA):

(1) $$\sigma \;=\;\sigma_{o}\;+\;K\;\dot{\gamma }{\;}^{n},$$

where, σ_o,_ K, and *n* represent yield stress (Pa), consistency coefficient (Pa∙s), and flow behaviour index (*n*), respectively [35].

### 2.7. Statistical Analysis

The data were analysed using a randomised complete block design, which incorporated the five unsalted products (FMWC 1.5, FMWC 1.9, FMWC 2.3, FMWC 3.0, and FMWC 4.0) and five salted products (FMWC 1.5s, FMWC 1.9s, FMWC 2.3s, FMWC 3.0s, and FMWC 4.0s). Analysis of variance (ANOVA) was carried out using the general linear model (GLM) procedure of SAS 9.3 (SAS Institute Inc., Cary, NC, USA) [36], where the effect of treatment (fermented milk-to-wheat ratio, and presence or absence of salt) on each response variable was determined. Tukey’s multiple-comparison test was used for paired comparison of treatment means, and the level of significance was determined at *p* < 0.05.

The R-3.2.2 software [37] was used to compute a Pearson correlation (*r*) between different compositional parameters of the dehydrated FMWCs and their characteristics on reconstitution, where significance was determined at *p* < 0.05, *p* < 0.01, and *p* < 0.001, by applying Student’s *t*-test to *r* with *n*-2 degrees of freedom (df), where *n* is the number FMWC treatments and their replicates.

## 3. Results

### 3.1. Properties of Dehydrated Fermented Milk–Wheat Composite

#### 3.1.1. Composition

The compositions of the salted and unsalted FMWCs are shown in Table 2. The pH and levels of fat, protein, starch, and salt in all FMWCs, apart from FMWC 4.0 and FMWC 4.0s, are within the respective ranges reported by Tamime et al. [18] for commercial Lebanese kishk, i.e., pH 3.5–4.12, 14.7–21.4% protein, 2.4–11.5% fat, 42–59% starch, and 0.05–4.5% NaCl. The concentrations of lactose (6.4–10.9%) and galactose (2.4–4.2%) in the unsalted FMWCs were markedly higher and lower, respectively, than those (0.56–2.86%, and 2.8–10.4%) found by Tamime et al. [18]. Differences in sugars most likely reflects variations in the types of cultures used for milk fermentation (e.g., *Streptococcus thermophiles* and *Lactobacillus delbrueckii* subsp. *bulgaricus*), their lactose fermenting ability, the ratio of fermented milk to cereal, and manufacturing procedure [8,38].

Increasing the FM:W ratio resulted in significant increases in the contents of fat, protein, lactose, salt, lactic acid and galactose, and reductions in the level of starch and TS. The reduction in pH of the reconstituted FMWC (5% TS) as the fermented milk-to-wheat ratio increased was consistent with the increase in lactic acid content, concomitant with the increase in the proportion of fermented milk.

The addition of salt (1%) had little effect on pH or composition, apart from salt, which increased from 0.4–0.7% in the unsalted products to 2.5–3.6% in the salted variants. The salt content of both the unsalted and salted FMWCs increased with FM:W ratio. The salt levels of the salted and unsalted FMWCs were within the range (0.95–4.48%) reported by Tamime et al. [18] for retail Lebanese kishk, and suggest varying rates of NaCl addition by local manufacturers, probably owing to tradition, preservation, and taste.

#### 3.1.2. Particle Size

The particle size of all FMWC was within the range (D_50_: 183–371 µm) reported by Salameh et al. [39] for retail Lebanese kishk from cow’s milk, goat’s milk, or mixtures of both. The particle size distribution of the FMWC products was unaffected by the FM:W ratio, as reflected by the similar D_10_, D_50_, and D_90_ values (Table 2). This suggests similar breaking (fracture) properties of the cake following drying and prior to size reducing and milling, irrespective of the FM:W ratio. Likewise, salt addition had little effect, except on the D_10_ value of the FMWC 1.5 product.

#### 3.1.3. Water Sorption Behaviour of Dehydrated Fermented Milk–Wheat Composites

**Figure 1:**
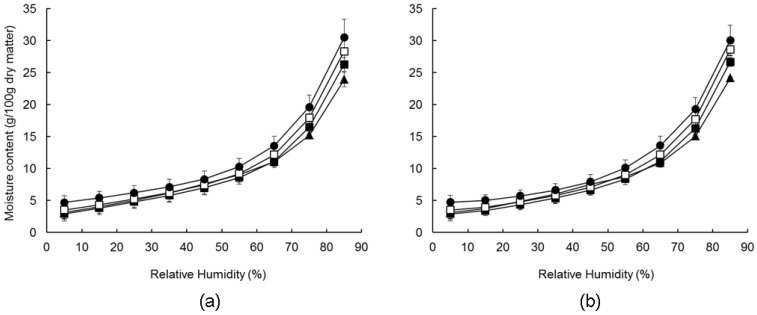
Sorption isotherms for unsalted fermented milk–wheat composites (FMWC) during desorption (**a**) and adsorption (**b**). The ratio of fermented milk-to-wheat was 1.5(▲), 2.3 (■), 3.0 (☐), or 4.0 (●). Presented values are the means of two replicate trials; error bars represent standard deviations of the mean.

The water desorption and adsorption isotherms of the unsalted FMWC products at 5–85% RH and 20 °C are shown in Figure 1; similar trends were obtained for the salted products (Supplementary Materials Figure S1). On initial equilibrium at 85% RH, the moisture content of all FMWC increased from 4–8 to 24–35%, depending on FM:W ratio and salt level (Table 2). Moisture uptake reflects the adsorption of water molecules to the surface of FMWC particles, either by their binding to the hydrophilic (charged and polar) groups of surface constituents through various types of interaction, such as hydrogen bonding to hydroxyl- and ionic- groups, or by capillary condensation [40,41]. The moisture content of all FMWC decreased on lowering the RH from 85 to 5% (Figure 1a), with the decrease being most pronounced in the 85–60% RH zone and more gradual thereafter. On increasing the RH from 5 to 85%, moisture increased in all FMWC to its original values, with the equilibrium values at each RH coinciding with those on desorption. The absence of hysteresis between the desorption and adsorption isotherms of the FMWC has also been reported for tarhana [42], and suggests that there were no changes in the particle structure during desorption, which would affect the accessibility and binding of water molecules on subsequent adsorption [43].

The equilibrium moisture content of the unsalted FMWC at 85% RH and 5% RH increased significantly with the FM:W ratio (Table 2). This trend is consistent with the concomitant increase in weight of lower molecular weight saccharides (e.g., lactose and galactose), which have a higher number of hydroxyl groups per unit weight than starch.

The equilibrium moisture content of the salted FMWCs at each RH was higher than that of the corresponding unsalted FMWCs, but only significantly so in the cases of FMWC2.3 and FMWC1.5.

### 3.2. Properties of Reconstituted Fermented Milk–Wheat Composites

#### 3.2.1. Gelatinization Temperature

**Figure 2:**
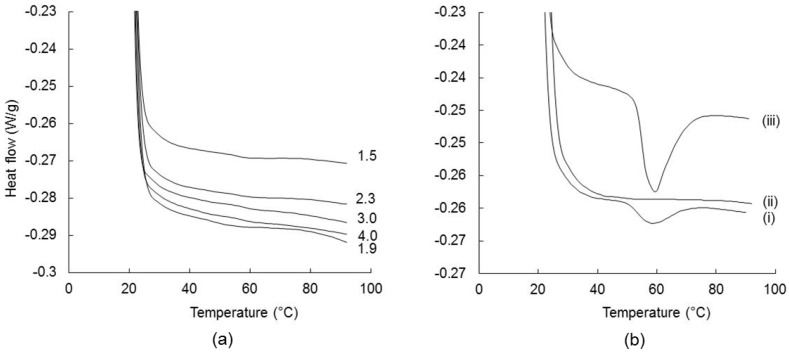
Differential scanning calorimetry (DSC) endotherms for (**a**) unsalted fermented milk–wheat composites (FMWC) with different ratios of fermented milk to wheat: 1.5, 1.9, 2.3, 3.0, or 4.0 as shown; and (**b**) non-parboiled wheat (i), parboiled wheat (ii), and native wheat starch (iii).

The gelatinization curves of the reconstituted unsalted FMWC (6.3% TS) are shown in Figure 2a. None of the FMWCs underwent gelatinization, as indicated by the absence of an endothermic phase transition DSC peak. Its absence suggests that parboiling of the cereal at 95–100 °C for 70 min, prior to its inclusion in the formulation of the FMWC, had an effect similar to pre-gelatinization of starch [44]. This is supported by the occurrence of an endothermic phase transition DSC peak at 60 °C on heating reconstituted non-parboiled wheat or native wheat starch, and the absence of such a peak from the corresponding DSC profile of the reconstituted parboiled wheat starch (Figure 2b). Similarly, Sittipod and Shi [45] reported that parboiling of rice starch at 110 °C for 20 min resulted in the loss of an endothermic DSC peak. Similar to pre-gelatinization treatment, parboiling coincides with a loss of birefringence and the melting of the crystalline regions of the starch granule [45,46].

A similar trend was found for the salted FMWCs (Supplementary Materials Figure S2).

#### 3.2.2. Water Holding Capacity

**Figure 3:**
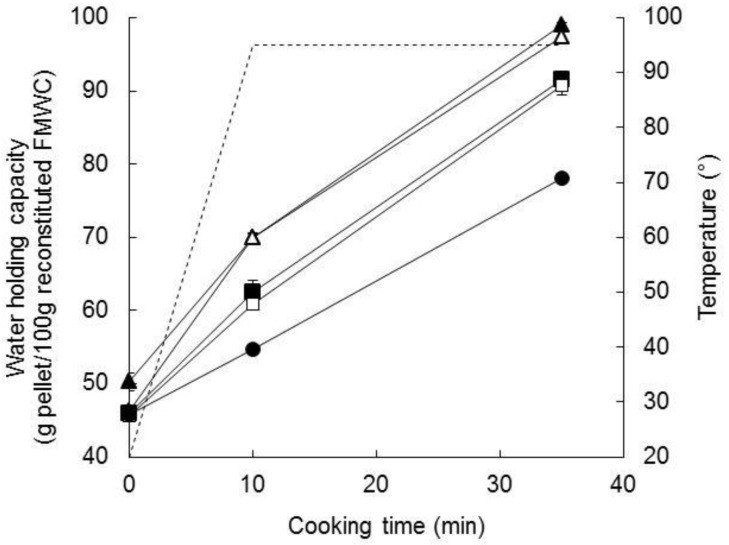
Changes in temperature (----) and water-holding capacity of unsalted fermented milk–wheat composites (FMWC), reconstituted to 16.7% total solids (solid lines) during cooking. The fermented milk-to-wheat ratio of the FMWC was 1.5 (▲), 1.9 (△), 2.3 (■), 3.0 (☐), or 4.0 (●). Presented values are the means of two replicate trials; error bars represent standard deviations of the mean.

The WHC of the reconstituted unsalted FMWCs (16.67% TS; 6.3–8.5% starch) increased during cooking (Figure 3). This trend is consistent with the hydration and swelling of the starch granules [46,47]. The value at the end of the holding period decreased with the FM:W ratio (*p* < 0.05), highlighting the central role of starch—which decreased concomitantly from 8.7 to 6.5%—in controlling water uptake on cooking the reconstituted FMWC. Hence, regression analysis indicated that WHC at 10 or 35 min correlated positively with the starch content (*R* = 0.89, df = 8).

The addition of salt during the formulation of the FMWC had little or no effect on WHC (Table 3).

#### 3.2.3. Pasting Behaviour

**Figure 4:**
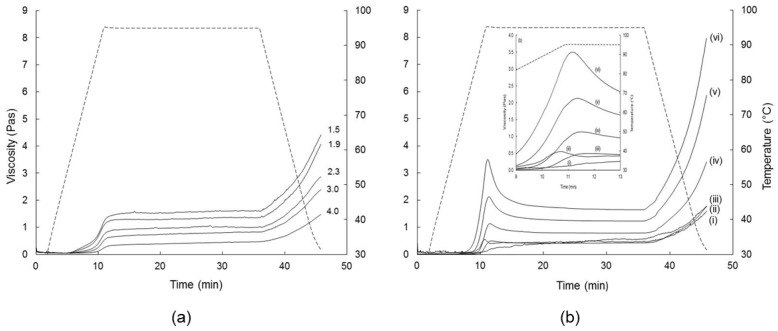
Pasting curves of reconstituted (**a**) unsalted fermented milk–wheat composites (FMWC) with different ratios of fermented milk to wheat: 1.5, 1.9, 2.3, 3.0, or 4.0, as shown; and (**b**) non-parboiled wheat (i), parboiled wheat (ii) and native wheat starch at levels of 6 (iii), 7 (iv), 8 (v), or 9 (vi) %, as shown. The broken line (----) shows temperature, while the inset in (b) shows the changes after heating to 95 °C in more detail.

The pasting curves of the reconstituted unsalted FMWCs on heating to 95 °C, holding at 95 °C, and cooling to 30 °C are shown in Figure 4a; similar curves were obtained for the salted FMWCs (Supplementary Materials Figure S3). The pasting curves of reconstituted native wheat starch (6–9% starch), non-parboiled wheat, and parboiled wheat (Figure 4b) were included as reference products.

Similar to the trend for WHC, the viscosity increased continuously with time, with the increases most pronounced during heating and cooling. The pasting profile of the FMWCs was similar to that of the parboiled wheat, but contrasted with that of non-parboiled wheat and native wheat starch, both of which displayed a peak viscosity on heating to 95 °C, or within 1 to 2 min of holding at 95 °C, after which viscosity decreased steeply during the next 5 min of holding, and thereafter more slowly during the remainder of the holding period. The absence of a peak pasting viscosity of the FMWCs is consistent with the results of previous studies on rice starch, which found that parboiling led to reductions of peak viscosity, breakdown viscosity, and setback viscosity, to an extent depending on the duration and temperature of parboiling [48,49,50,51,52]. The results suggest partial alteration or rupture of the starch granule during parboiling and subsequent milling, which reduces pasting viscosity during subsequent pasting [44,53,54].

**Figure 5:**
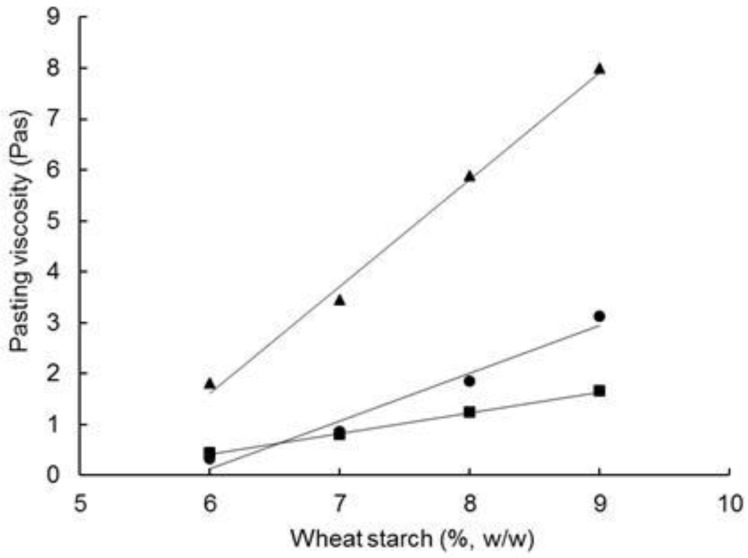
Influence of starch content on the viscosity of a wheat starch suspension after different times during pasting: after heating to 95 °C over 10 min, V_95_ (●); holding at 95 °C for 25 min, V_h_ (■); and cooling to 30 °C, V_c_ (▲).

Increasing the FM:W ratio coincided with significant reductions in V_95_, V_h_, V_c_, and setback viscosity of the reconstituted FMWCs (Table 3, Figure 4a). Analogously, Eliasson [55] found that the storage modulus, G′, of hot (95 °C) suspensions of native wheat, maize, or corn starch increased with the starch content in the range of 7.5 to 12.5% (*w*/*w*). These trends highlight the importance of starch content—which decreased concomitantly from 8.7 to 6.5% with the FM:W ratio—on water immobilization and viscosity development; hence, the WHC at 10 and 35 min correlated positively with V_95_, V_h_, V_c_ (*R* = 0.91–0.98, df = 8). The influence of starch content as a viscosity modulator is supported by the linear increase in V_95_, V_h_ and V_c_ of native wheat starch on increasing starch content in the range 6.0 to 9.0% (Figure 5). However, differences in the concentrations of other materials such as protein, fat, salt, lactose, and lactic acid in the reconstituted FMWCs are also likely to affect the pasting behaviour of starch [56,57,58,59].

Increasing NaCl concentration in the range of 1.2 to 6% (*w*/*w*) has been reported to increase gelatinization temperature and the storage modulus (G′) of heated (95 °C) starch suspensions [60,61]. However, the current results show little or no effect of added salt on the pasting behaviour of the reconstituted FMWC (Table 2). The overall lack of an effect most likely reflects the low salt concentration in the reconstituted FMWCs (0.07–0.6% *w*/*w*).

#### 3.2.4. Rheological Properties.

**Figure 6:**
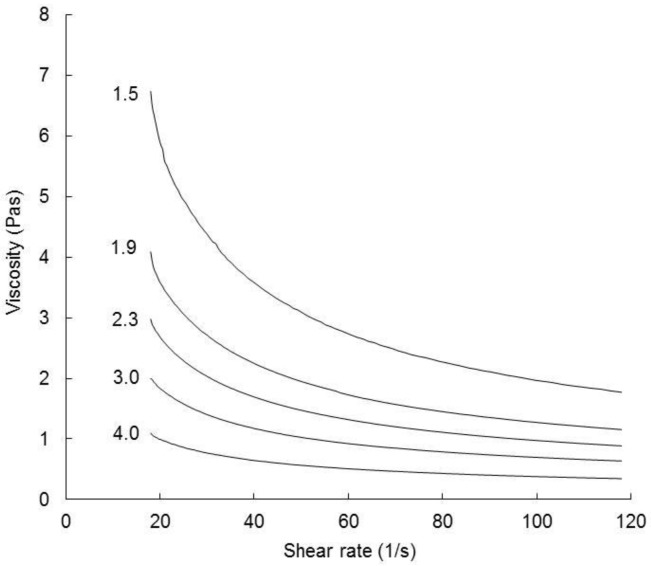
Flow curves of unsalted fermented milk–wheat composites (FMWC), reconstituted to 16.7% total solids, at 60 °C. The fermented milk-to-wheat ratio of the FMWC was 1.5, 1.9, 2.3, 3.0, or 4.0, as shown.

Following holding at 95 °C, the reconstituted FMWCs were cooled to 60 °C and subjected to a shear rate sweep from 18 to 120 s^−1^. The shear rate versus shear stress data of all FMWCs fitted to the Herschel–Bulkley model (*R* > 0.99). All exhibited a yield stress and shear thinning behaviour (*n* < 1) (Figure 6), reflecting the presence of internal network, most likely a closepacked suspension of swollen starch granules, which was disrupted during shearing.

Increasing the FM:W ratio coincided with significant reductions in σ_0_, K, and viscosity over the entire shear rate range of the reconstituted unsalted, or salted FMWCs (Table 3), but did not affect the extent of shear thinning (*n*). Similarly, Aguilar-Raymundo and Vélez-Ruiz [62] found that the viscosity of custard over the shear rate range of 2–30 s^−1^ increased with content of chickpea flour in the range of 8.3 to 11.3 (*w*/*w*). Hence, regression analysis indicated that σ_0_, K, and η_120s-1_ correlated positively with starch content (*R* = 0.88, 0.9, and 0.84, respectively; df = 8).

The addition of salt during formulation had little to no effect on flow behaviour properties (Table 3).

## 4. Conclusions

Increasing the ratio of fermented milk (FM)-to-wheat (W) in dehydrated fermented milk wheat composites (FMWCs) from 1.5 to 4.0 resulted in lower starch content and pH, as well as higher levels of protein, fat, lactose, lactic acid, and salt. These changes coincided with significant changes in the consistency of the reconstituted FMWC (16.7% TS), which varied from porridge-like at FM:W ratios of 1.5–1.9 to soup-like at ratios of 3.0–4.0. Increasing the FM:W ratio led to notable reductions in water holding capacity, viscosity during pasting (heating and holding at 95 °C), and viscosity setback during cooling, The reconstituted FMWC exhibited a yield stress (σ_0_) and underwent pseudoplastic flow on shearing. Increasing the FM:W ratio led to reductions in σ_0_, consistency coefficient, and viscosity. The current study provides new information on how varying the proportion of fermented milk affects the compositional and reconstitution behaviour of FMWCs. The findings should provide valuable insight into the design and innovation of milk (fermented milk)-cereal based food composites, which combine the nutritive, techno-functional, and flavour properties of two major food groups. It would be of interest in future studies to investigate the impacts of the FM:W ratio on the sensory and digestibility characteristics of FMWCs.

## Figures and Tables

**Table 1:** Ingredients used in formulation of salted and unsalted dehydrated fermented milk–wheat composites (FMWC) ^1,2^.

Ingredient Used (%, *w*/*w*)	Unsalted FMWC					Unsalted FMWC				
	1.5	1.9	2.3	3.0	4.0	1.5s	1.9s	2.3s	3.0s	4.0s
FM	60	65	70	75	80	59.4	64.4	69.3	74.3	79.2
Parboiled wheat	40	35	30	25	20	39.6	34.7	29.7	24.8	19.8
Salt	0	0	0	0	0	1	1	1	1	1

^1^ FM: fermented milk. ^2^ The unsalted FMWC 1.5, FMWC 1.9, FMWC 2.3, FMWC 3.0, FMWC 4.0 had fermented milk-to-wheat ratios of 1.5, 1.9, 2.3, 3.0, and 4.0, respectively; the ratio of fermented milk-to-wheat was similarly varied in the salted FMWC. FMWC: fermented milk–wheat composites.

**Table 2:** Effect of ratio of fermented milk-to-parboiled wheat (FM:W) on the characteristics of fermented milk-wheat composites (FMWC) ^1–4^.

Characteristic	Unsalted FMWC						Salted FMWC					
	FM:W						FM:W					
	1.5	1.9	2.3	3.0	4.0	SED ^2^	1.5s	1.9s	2.3s	3.0s	4.0s	SED ^2^
Composition												
Total Solids (%, *w*/*w*)	95.9 ^aA^	96.0 ^aA^	95.2 ^bA^	94.6 ^cA^	92.3 ^dA^	0.23	95.7 ^aA^	96.0 ^aA^	95.4 ^aA^	94.1 ^aA^	92.0 ^bA^	0.45
Fat (%, *w*/*w*)	3.7 ^eA^	4.2 ^dA^	4.6 ^cA^	5.2 ^bA^	5.9 ^aA^	0.26	3.7 ^eA^	4.1 ^dA^	4.5 ^cA^	5.1 ^bA^	5.8 ^aA^	0.30
Protein (%, *w*/*w*)	15.2 ^cA^	15.9 ^bcA^	16.7 ^bcA^	17.9 ^abA^	18.9 ^aA^	0.29	14.2 ^cA^	15.4 ^bcA^	16.9 ^abA^	17.3 ^abA^	18.6 ^aB^	0.47
Starch (%, *w*/*w*)	52.0 ^aA^	48.8 ^aA^	47.1 ^abA^	42.3 ^bcA^	38.9 ^cA^	1.06	51.1 ^aA^	49.9 ^aA^	45.8 ^abA^	41.2 ^bcA^	37.6 ^cA^	0.94
Lactose (%, *w*/*w*)	6.4 ^aA^	7.4 ^aA^	8.0 ^aA^	9.7 ^aA^	10.9 ^aA^	3.23	6.6 ^bA^	7.9 ^abA^	8.7 ^abA^	9.9 ^abA^	11.4 ^aA^	2.86
Galactose (%, *w*/*w*)	2.4 ^cA^	2.9 ^bA^	3.3 ^bA^	3.8 ^aA^	4.2 ^aA^	0.06	2.2 ^cA^	2.8 ^bcA^	3.2 ^abcA^	3.5 ^abA^	4.2 ^aA^	0.19
Lactic acid (%, *w*/*w*)	2.7 ^cA^	3.0 ^cA^	3.6 ^bA^	3.8 ^abA^	4.2 ^aA^	0.25	2.4 ^cA^	2.9 ^bcA^	3.2 ^bA^	3.5 ^abA^	4.0 ^aA^	0.19
Salt (%, *w*/*w*)	0.42 ^eB^	0.49 ^dB^	0.55 ^cB^	0.61 ^bB^	0.70 ^aB^	0.005	2.50 ^eA^	2.68 ^dA^	2.95 ^cA^	3.50 ^bA^	3.61 ^aA^	0.01
pH	4.1 ^aA^	4.0 ^aA^	3.9 ^bA^	3.9 ^bA^	3.9 ^bA^	0.03	4.2 ^aAA^	4.0 ^bA^	4.0 ^bcA^	4.0 ^bcA^	3.9 ^cA^	0.05
Particle diameter ^3^												
D_10_ (µm)	42 ^aA^	36 ^aA^	36 ^aA^	36 ^aA^	36 ^aA^	1.36	34 ^aB^	34 ^aA^	33 ^aA^	35 ^aA^	41 ^aA^	2.36
D_50_ (µm)	208 ^aA^	195 ^bA^	186 ^bcA^	185 ^bcA^	180 ^cA^	1.73	192 ^aA^	196 ^aA^	186 ^aA^	182 ^aA^	195 ^aA^	4.95
D_90_ (µm)	501 ^aA^	493 ^abA^	477 ^abA^	482 ^abA^	475 ^bA^	4.12	472 ^aA^	496 ^aA^	482 ^aA^	475 ^aA^	491 ^aA^	7.28
Water sorption (g water/100 g dm)												
At 85% RH ^4^	23.9 ^cB^	25.0 ^bcA^	26.3 ^bcB^	28.3 ^abA^	30.5 ^aA^	1.35	29.01 ^aA^	29.7 ^aA^	32.8 ^aA^	35.4 ^aA^	34.7 ^aA^	1.33
At 5% RH	3.1 ^bA^	2.8 ^bA^	2.9 ^bA^	3.5 ^bA^	4.7 ^aA^	0.87	2.8 ^aA^	3.2 ^aA^	3.2 ^aA^	3.6 ^aA^	4.6 ^aA^	0.62

**^a^**^–d^ Values within a row relating to unsalted or salted FMWC and not sharing a common lower-case superscript differ significantly (*p* < 0.05) for effect of the ratio of fermented milk to wheat (1.5, 1.9, 2.3, 3.0 and 4.0). **^A^**^,B^ Values within a row relating to any specific fermented milk–wheat ratio and not sharing a common upper-case superscript differ significantly (*p* < 0.05) for effect of salting;. ^1^ Presented data are the mean values of two replicate trials. ^2^ SED: standard error of difference between means. ^3^ D_10_, D_50_, and D_90_: the particle diameters of <10%, <50%, and <90% of the powder particles, respectively. ^4^ RH: relative humidity. FMWC, fermented milk-wheat composite.

**Table 3:** Effect of ratio of fermented milk-to-parboiled wheat (FM:W) on the characteristics of reconstituted fermented milk–wheat composites (FMWC) ^1,2^.

Characteristic	Unsalted FMWC						Salted FMWC					
	FM:W						FM:W					
	1.5	1.9	2.3	3.0	4.0	SED ^1^	1.5s	1.9s	2.3s	3.0s	4.0s	SED ^1^
WHC (g/100 g) ^2^												
At 0 min	50.3 ^a^	46.3 ^b^	45.9 ^b^	45.7 ^b^	45.9 ^b^	0.58	-	-	-	-	-	-
At 10 min	70.0 ^a^	69.9 ^a^	62.5 ^b^	60.9 ^b^	54.7 ^c^	0.64	-	-	-	-	-	-
At 35 min	99.0 ^aA^	97.4 ^aA^	91.5 ^bA^	90.8 ^bA^	78.0 ^cA^	0.58	94.7 ^aA^	98.6 ^aA^	97.2 ^aA^	90.5 ^aA^	72.2 ^bA^	1.27
Pasting Characteristics ^2^												
V_95_ (Pa·s)	1.17 ^aA^	1.04 ^aA^	0.71 ^bA^	0.50 ^bA^	0.23 ^cA^	0.04	0.56 ^bcB^	0.92 ^aA^	0.68 ^abA^	0.31 ^cA^	0.25 ^cA^	0.06
V_h_ (Pa·s)	1.61 ^aA^	1.36 ^abA^	1.02 ^abcA^	0.83 ^bcA^	0.46 ^cA^	0.09	0.98 ^abA^	1.40 ^aA^	1.05 ^abA^	0.65 ^bA^	0.47 ^bA^	0.10
V_c_ (Pa·s)	4.42 ^aA^	4.07 ^abA^	2.87 ^bcA^	2.39 ^cdA^	1.47 ^dA^	0.20	2.95 ^abA^	4.20 ^aA^	3.19 ^abA^	2.07 ^bA^	1.47 ^bA^	0.40
SBV (Pa·s)	2.81 ^aA^	2.71 ^aA^	1.85 ^bA^	1.57 ^bcA^	1.01 ^cA^	0.15	1.98 ^abA^	2.80 ^aA^	2.14 ^abA^	1.42 ^abA^	0.99 ^bA^	0.30
Rheology ^2^												
σ_o_	67.2 ^aA^	36.6 ^bA^	24.0 ^bcA^	18.0 ^cA^	6.5 ^dB^	3.39	71.8 ^aA^	35.2 ^bA^	24.1 ^bcA^	14.0 ^cdA^	8.0 ^dA^	2.82
k (Pa·s^n^)	10.2 ^aA^	6.3 ^bA^	6.0 ^bA^	2.8 ^cA^	2.7 ^cA^	0.54	6.3 ^aA^	4.70 ^bA^	3.7 ^cB^	3.2 ^cdA^	2.64 ^dA^	0.11
*n* (-)	0.60 ^A^	0.59 ^aA^	0.57 ^aA^	0.63 ^aA^	0.53 ^aA^	0.01	0.65 ^aA^	0.61 ^aA^	0.61 ^aA^	0.60 ^aA^	0.49 ^aA^	0.05
η at 120 s^−1^ (Pa·s)	1.7 ^aA^	1.21 ^abA^	0.85 ^abA^	0.64 ^abA^	0.34 ^bA^	0.24	1.61 ^aA^	1.08 ^bA^	0.85 ^bcA^	0.51 ^cdA^	0.36 ^dA^	0.06

^a–d^ Values within a row relating to unsalted or salted FMWC and not sharing a common lower-case differ significantly (*p* < 0.05) for effect of ratio of fermented milk to wheat. ^A,B^ Values within a row relating to any specific fermented milk–wheat ratio and not sharing a common upper-case superscripted letter differ significantly (*p* < 0.05) for effect of salting; salt (1%, *w*/*w*) was added during the formulation of the salted FMWC, but not in the case of the unsalted FMWC. ^1^ Presented data are the mean values of two replicate trials; SED: standard error of differences between means. ^2^ WHC: water holding capacity before heating (0 min), after cooking to 95 °C (10 min), and after holding at 95 °C for 25 min (35 min); V_95_, V_h_, and V_c_ refer to viscosity after heating to 95 °C, holding at 95 °C for 25 min, and cooling to 30 °C, respectively; SBV: setback viscosity;$\sigma_{o}$ *k*, *n*, and η_120 s_^−1^ denote yield stress, consistency index, flow behaviour index, and final viscosity at 120 s^−1^ after shearing from 18 to 120 s^−1^ at 60 °C, respectively; -: not measured; FMWC, fermented milk–wheat composite.

## Supplementary Materials

The following are available online at http://www.mdpi.com/2304-8158/7/7/113/s1, Figure S1: Sorption isotherms for salted dehydrated fermented milk-wheat composites (FMWC) during desorption (a) and adsorption (b): The ratio of fermented milk-to-wheat composites was 1.5 (▲), 2.3 (■), 3.0 (☐) or 4.0 (●). Presented values are the means of two replicate trials; error bars represent standard deviations of the mean, Figure S2: DSC endotherms for salted (s) dehydrated fermented milk–wheat composites (FMWC) with different ratios of fermented milk to wheat: 1.5, 1.9, 2.3, 3.0 or 4.0, Figure S3: Pasting curves of salted (s) dehydrated fermented milk-wheat composites (FMWC) with different ratios of fermented milk to wheat: 1.5, 1.9, 2.3, 3.0 or 4.0.
