# Supplementary material for: The Proportion of Fermented Milk in Dehydrated Fermented Milk–Parboiled Wheat Composites Significantly Affects Their Composition, Pasting Behaviour, and Flow Properties on Reconstitution
Source: Foods. 2018 Jul 14;7(7):113. doi: 10.3390/foods7070113 (PMC6068679; doi:10.3390/foods7070113)
Supplement: Supplementary file 1 [file foods-07-00113-s001.pdf]

# Supplementary Materials: The Proportion of Fermented Milk in Dehydrated Fermented Milk-Parboiled Wheat Composites Significantly Affects their Composition, Pasting Behaviour and Flow Properties on Reconstitution

**Ashwini V. Shevade [1], Yvonne C. O' Callaghan [2], Nora M. O' Brien [2], Tom P. O' Connor [2], Timothy P. Guinee [1,*]**

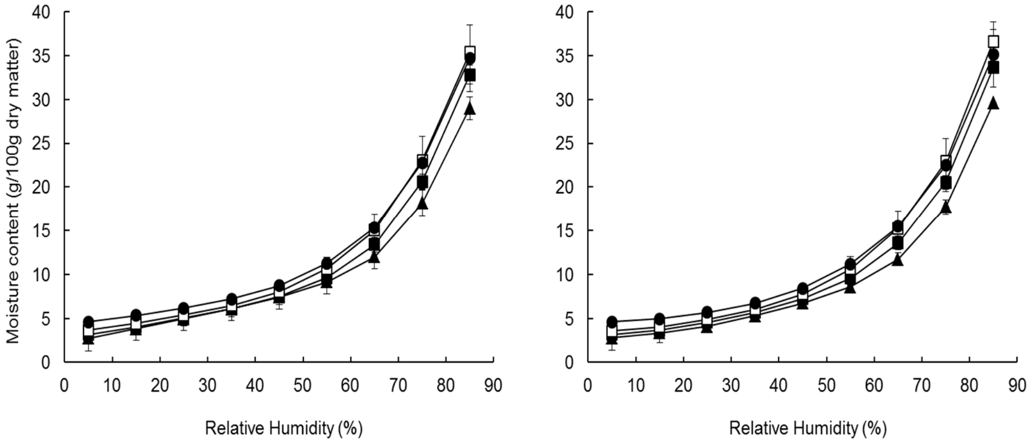


(a) (b)

**Figure S1.** Sorption isotherms for salted dehydrated fermented milk-wheat composites (FMWC) during desorption (a) and adsorption (b): The ratio of fermented milk-to-wheat composites was 1.5(▲), 2.3 (■), 3.0 (□) or 4.0 (●).Presented values are the means of two replicate trials; error bars represent standard deviations of the mean.

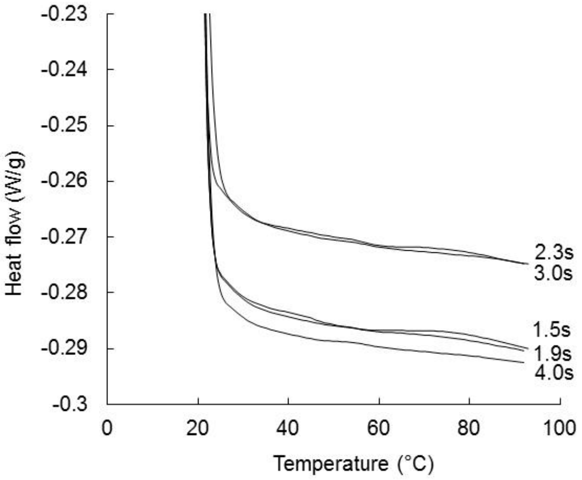


**Figure S2.** DSC endotherms for salted (s) dehydrated fermented milk-wheat composites (FMWC) with different ratios of fermented milk to wheat: 1.5, 1.9, 2.3, 3.0 or 4.0.

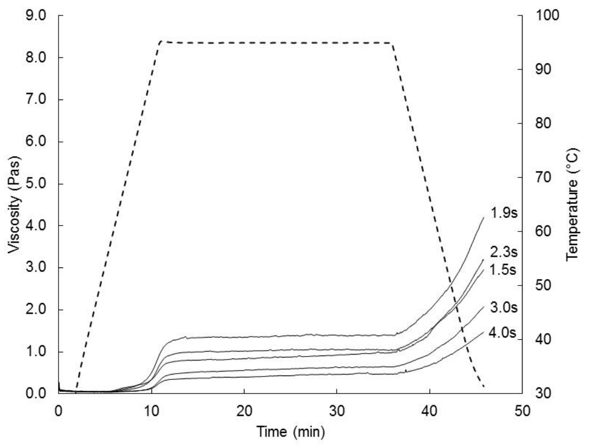


**Figure S3.** Pasting curves of salted (s) dehydrated fermented milk-wheat composites (FMWC) with different ratios of fermented milk to wheat: 1.5, 1.9, 2.3, 3.0 or 4.0.
